# Microbial Contamination of Bedding Material: One Health in Poultry Production

**DOI:** 10.3390/ijerph192416508

**Published:** 2022-12-08

**Authors:** Bianca Gomes, Pedro Pena, Renata Cervantes, Marta Dias, Carla Viegas

**Affiliations:** 1 H&TRC—Health & Technology Research Center, ESTeSL—Escola Superior de Tecnologia e Saúde, Instituto Politécnico de Lisboa, 1990-096 Lisbon, Portugal; 2 CE3C—Center for Ecology, Evolution and Environmental Change, Faculdade de Ciências, Universidade de Lisboa, 1749-016 Lisbon, Portugal; 3 Public Health Research Centre, NOVA National School of Public Health, Universidade NOVA de Lisboa, 1600-560 Lisbon, Portugal; 4 Comprehensive Health Research Center (CHRC), 1600-560 Lisbon, Portugal

**Keywords:** microbial contamination, poultry bedding material, mycotoxins, microbial resistance

## Abstract

In poultry farms, the mixture of bedding material, chicken excrement, and feathers seems to play an important role in pathogen development which may contribute to a potential risk of zoonosis, spreading the disease through the food chain. The purpose of this study was to analyze microbial contamination in bedding material and other matrices as well as potential antimicrobial resistances in chicken production facilities, and also to identify the sampling techniques and assays used. This study evidences the available data published, following the PRISMA methodology. Among the environmental samples, surface swabs were frequently used as a passive sampling technique. Morphological identification was performed in all studies. From all the matrices, the bedding material was the most contaminated. Most studies focused on bacterial contamination, with *Salmonella* sp. and *Campylobacter* sp. being commonly reported and three studies evidenced fungal contamination, being *Penicillium* sp.- and *Aspergillus* sp.-dominant. Mycotoxin assessment was only performed in one study, being identified in all bedding samples. The screening for bacteria resistance evidenced bacteria multidrug resistance; however, fungal susceptibility to azoles was not assessed in any of the analyzed studies. Briefly, this review evidences the microbial contamination in poultry facilities, emphasizing animals’ bedding as a potential source of contamination. Additionally, this study contributes to a sampling and analysis protocol proposal to assess the microbial contamination in this setting. Additionally, the knowledge gaps identified highlight the need of further research regarding microbial contamination and toxicological potential on animals’ bedding in order to mitigate the exposure in poultry pavilions.

## 1. Introduction

Over the last few years, the consumption of animal-source food has led to the intensification of livestock production systems [1]. Such demands along with changing management practices influence the distribution and intensity of parasite infections, being linked with zoonotic diseases [1]. Poultry production is mostly confinement structures densely stocked with birds [2]. Thus, maintaining the ideal microclimate and animal hygiene conditions may entail some challenges [3]. Despite mechanical ventilation systems in order to maintain birds’ health, microorganisms from animals’ bedding are easily accumulated and aerosolized [2].

Feces, leftover food, bedding material, and feathers that characterize poultry litter are a high-quality, low-cost organic soil fertilizer that boosts crop quality and productivity, which explains its extensive use as manure around the world [4,5]. A proper bedding material, in terms of chemical and physical features as well as microbial counts, is a requirement for efficient broiler production [6], and wood-based beddings were associated with an improvement in birds’ performance [7].

Aside from its organic composition, the material used as a bed for animals can harbor a variety of pathogens such as viruses, bacteria, parasites, and fungi [8]. Additionally, a mixture of bedding materials, chicken excrement, and feathers seems to influence pathogen development [9]. Microbiological contamination of poultry farm air, including microbial composition, has been examined and analyzed in depth [2,3,10]. In fact, focusing on the rise of foodborne outbreaks [11], bacteria were found in animals’ bedding, with some *Salmonella* isolates showing antimicrobial resistance. Microbial pathogens are a major concern in the food industry [9]; consequently, food safety and security might be affected by litter quality [12].

From the poultry feed, air, and bedding material collected, fungal species potentially pathogenic for birds (*Aspergillus* sp. and *Candida albicans*) were recovered [2]. Indeed, a significant positive correlation was found between the fungal contamination found in air and animals’ bedding (pine shavings, straw, wood shavings, and wood shavings with rice hulls), suggesting that the material used as a bed is a contamination source for indoor fungal contamination [10].

Microbial counts are mostly controlled by ventilation system efficiency and air dustiness [3], and evidence suggests that broiler and animal bedding are the main sources of inside and outside air and soil pollution [3]. These findings should be considered as occupational and public health concerns since microbial aerosolization may result in long distance transport of potentially pathogenic fungi, and coliform bacteria to the surrounding area [3].

In what concerns chicken production, another concern relies on the intensive use of antibiotics either as growth promoters [13] or for prophylactic purposes [14]; there is also a risk of transmission of multidrug-resistant bacteria [8] and fungi [15]. Animals’ bedding preparation is one of the duties that exposes poultry workers to more dust [16] and fungi and their metabolites, such as volatile organic compounds (VOC) and mycotoxins [17,18]. Bedding material rich in keratinous components is plowed into agricultural soils after being utilized and removed from chicken farms. This might entail public health risks due to the spread of keratinophilic and toxigenic fungi already detected in bedding material samples [10]. Additionally, this method could be hazardous to the soil ecosystem, as well as to animals [10,19].

Chicken bedding material may be an underestimated source of antimicrobial resistance transmission towards animals, humans and the environment, emphasizing the current need for a One Health approach and antimicrobial resistance (AMR) surveillance concerning this subject [4]. The “One Health” concept involves collaborative efforts of multiple disciplines in order to reach the optimal health for people, animals and the environment [20]. It recognizes that human health is associated with the health of both animals and the environment [9]. Thus, when applying the One Health approach in a given setting, the health of humans, animals and the surrounding environment must be considered [9], including the risk factors associated with the farm environment, food industry and surrounding household settings [20]. The importance of food safety for public health has already been mentioned, and research efforts have been focused on animal health or production practices [20]. Recently, the literature has focused on bacteria contamination in livestock production [11,21,22,23]. However, less is known regarding the microbiological safety patterns and antimicrobial resistance from fungal contamination [10], although *Aspergillus*-resistant isolates were already isolated from different environmental matrices [24,25,26,27,28].

In the scope of the “One Health” approach, there is scarce information regarding poultries’ bedding material as a potential source of microbial contamination affecting animals, workers, and consumers’ health. Thus, this study aimed to perform a systematic review to provide a broad overview of the state of the art in the developed subject, describing the microbiological contamination found in the bedding materials used in poultry production and indicating which parameters and methods were applied to perform the microbial exposure assessment in this setting. This study’s results will contribute to a sampling and analysis protocol proposal aiming to assess microbial exposure in this indoor environment.

## 2. Materials and Methods

### 2.1. Registration

The Preferred Reporting Items for Systematic Reviews (PRISMA) checklist [29] was completed (Table S1—Supplementary Material).

### 2.2. Search Strategy and Inclusion and Exclusion Criteria

This study followed the PRISMA methodology of the available data published between 1 January 2000 and 1 January 2022. The search terms aimed to identify studies performed on poultry facilities. The databases chosen were PubMed (https://pubmed.ncbi.nlm.nih.gov/ (accessed on 15 January 2022)), Web of Science (www.webofscience.com (accessed on 15 January 2022)), and Scopus (https://www.scopus.com/ (accessed on January 2022)), and the selected terms were “Microbial contamination” and “Poultry litter”. Searches were carried out in English and articles that did not fulfill the inclusion criteria were not subjected to additional review (but some of them were used for introduction and discussion sections) (Table 1).

### 2.3. Studies’ Selection and Data Extraction

The selection of the articles was performed in three rounds through the Rayyan—Intelligent systematic review application. The first round was performed by one investigator (BG) and consisted of a screening of all titles in order to exclude papers that were duplicated or unrelated to the subject. The selected papers were then added to Rayyan for further analysis. The second round consisted of a screening of all abstracts and was performed by two investigators (BG and MD). In the third round, the full texts of all potentially relevant studies were reviewed considering the inclusion and exclusion criteria. Potential divergences in the selection of the studies were discussed and resolved by four investigators (BG, MD, RC, and PP). Data extraction was then performed by RC. Additionally, it was reviewed by BG. The following information was manually extracted: (1) databases, (2) title, (3) country, (4) occupational environment, (5) sampling methods, (6) analytical methods, (7) other analyzed matrixes, (8) main findings, and (9) references.

### 2.4. Quality Assessment

The assessment of the risk of bias was conducted by two investigators (BG and CV). Within each article, an evaluation of the risk of bias was performed across three parameters divided as key criteria (sampling methods and analytical methods) and other criteria (data related to microorganisms’ metabolites). The risk of bias for each parameter was evaluated as “low”, “medium”, “high”, or “not applicable”. The articles for which all the key criteria and most of the other criteria were characterized as “high” were excluded.

## 3. Results

**Figure 1:**
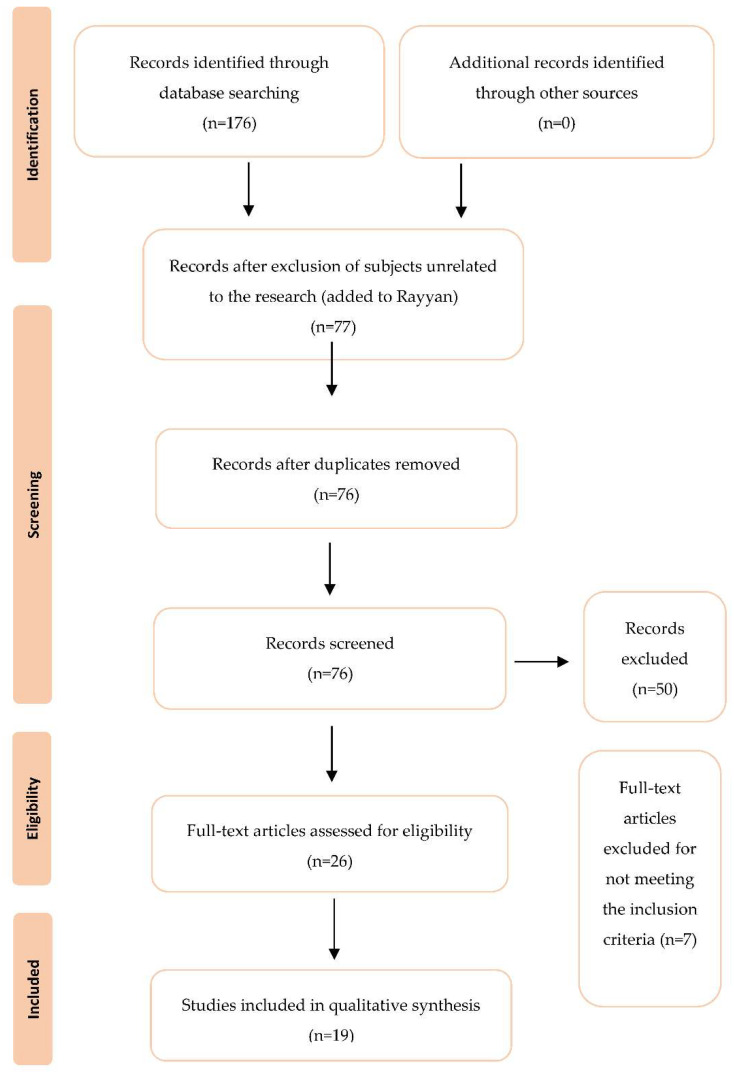
PRISMA based selection of articles.

Figure 1 shows the flow diagram for selecting the studies. Initially, the database search yielded 176 studies, from which 77 abstracts were analyzed and 26 full texts were reviewed for eligibility. A total of seven studies were rejected for not fulfilling the inclusion and exclusion criteria, primarily because they were not related to litter samples and contamination regarding poultry production. A total of 19 papers on litter contamination in poultries were selected.

Characteristics of the Selected Studies

The selected studies and their main characteristics are described in Table 2. Of the reviewed studies (*n* = 19), seven were conducted in the The United States of America [11,30,31,32,33,34,35], four in Europe (one in Portugal [10], one in France [2], one in Lithuania [36], and one in Bulgaria [3]), six in Africa (one in Argelia [37], one in Kuwait [22], two in Egypt [23,38], and two in Cameroon [4,21]), one in Asia (Korea [39]), and one in the Middle East (Iran [6]). Most of the studies were conducted in poultry farms (10 out of 19–53%) [10,11,21,29,31,33,35,36,37,38], four (21%) in big poultry farms [3,4,22,30], four (21%) in small poultry farms [2,5,32,34], and one in a big duck farm [39].

The sampling method used in all studies (100%) was animals’ bedding collection [2,3,4,6,10,11,21,23,30,31,32,33,34,35,36,37,38,39]. Eight out of nineteen studies (42%) specified the type of bedding material, from which four used pine shavings [10,30,35,38], two used straw [2,10], three used wood shavings [4,6,10], three used rice hulls [10,35,37], one used cow dungs, shredded paper barley stalks and a mixture of these materials with wood shavings and rice hulls [6].

All the 19 studies analyzed other matrixes: swabs—seven [22,23,30,32,36,38,39] (four cloacal/caeca [23,30,36,38], four drinker swabs [22,30,32,36], three feed storage [30,32,39], three attendants’ hand/shoes [23,30,38], three walls/floor [22,30,39], one ventilation fan [30], one feather [30], and one door handle [30]), feed—six [2,21,22,23,30,37]; water—five [2,21,22,23,37,38]; chicken carcasses—two [21,23]; one paper tray liner [22]; one dust [39]; and one shoe covering; air—four through impactation [2,10,22,30], one through the impinger method [33], one through the sedimentation method [3] and one through filtration [32].

Regarding contamination characterization in the 19 studies, 16 studies focused on bacterial contamination [3,4,11,21,22,23,30,31,32,33,34,35,36,37,38,39], and 3 studies on fungal contamination [2,6,10]. Nine studies detected *Salmonella* sp. [4,11,22,23,30,34,35,37,39], five detected *Campylobacter* sp. [31,32,34,36,38], and four detected *Staphylococci* sp. [31,33,34,35]. Three studies detected *Penicillium* sp. [2,6,10], *E. coli* [4,34,37], and Enterococci [31,34,35]. Two studies reported *Listeria* sp. [21,28], *Aspergillus flavus* [2,10], *Scopulariopsis* sp. [2,10], and *Clostridium perfringens* [34,35]. Other studies reported *Alternaria* sp. [10], *Cladosporium* sp. [10], *Aspergillus* sp. [6], *Aspergillus fumigatus* [10], *Aspergillus versicolor* [10], *Aspergillus niveus* [10], *Trichosporon* sp. [10], *Absidia corymbifera* [2], *Candida* sp. [2], *Lactobacilli* sp. [31], *Mucor* sp. [6], *Geothricum* sp. [6], and *Rhizopus* sp. [6]. 

Concerning the analytical methods, all studies used culture-based methods [2,3,4,6,10,11,21,22,23,30,31,32,33], and one study also assessed physical and chemical parameters [11], ten used molecular tools [2,11,23,31,33,34,35,36,38,39], and four serotyped their samples [11,22,23,39]. All the 19 studies carried out morphological identification [2,3,4,6,10,11,21,22,23,30,31,32,33,34,35,36,37,38,39], 8 performed biochemical tests [4,21,23,30,31,32,33,38], 8 performed susceptibility tests [4,11,21,30,33,34,35,39], and 1 assessed mycotoxin [31].

From all sampling matrixes, chickens’ bedding material was reported to be contaminated in 18 out of 19 studies [2,3,4,6,10,11,21,22,23,30,38], being the most contaminated matrix in 12/19 studies [3,4,6,23,30,31,32,33,34,35,37,39]. Four studies presented contamination in feed [2,32,37,38], being the most contaminated matrix in three out of these four studies, and drinkers [32,36,37,38], while two showed contamination in air samples [2,32]. One study showed contamination in the attendants’ hands swabs [38], in broiler cloacae swabs [30], wall swabs, and floor swabs [37].

Mycotoxin assessment was only performed in one study [35], where eight different mycotoxins were targeted with zearalenone being detected in all animals’ bedding samples.

Eight of the studies that performed susceptibility tests or biomedical tests for resistance detection reported multidrug resistance [4,11,21,30,33,34,35,39].

## 4. Discussion

The presence of microbial pathogens in several stages of poultry and meat product processing has already been suggested [10]. Poultry facilities are frequently considered a source of human contamination [38], with litter being a source of human pathogens [11]. 

The mixture of bedding materials, chicken excrement, and feathers seems to play an important role in pathogen development [10]. Indeed, the material used as animals’ bedding may influence a bird’s performance since it affects the microbiological colonization of the animal [40]. Consequently, the microbial presence in poultry pavilions may increase the potential risk of zoonosis. As a matter of fact, when it comes to turkey’s health, fungal diseases caused by fungal species belonging to the genus *Aspergillus* are of critical importance [2]. Thus, animals’ bedding material analysis is vital to evaluate occupational and public health risks [10]. Foodborne pathogen diseases are usually self-limited; however, some can lead to serious illnesses [38].

A while ago, a case of aspergillosis was reported in turkeys’ flocks using wood shavings as animal bed material [41]. Wood shavings seem to present high fungal counts [10]. Additionally, there is some evidence pointing out the potential of shredded paper to host *Aspergillus* as a hazardous fungus [10]. These findings might draw some attention in settings where these materials are used in animal bedding. In fact, pine shavings were recurrent in some studies [10,30,35,38]. Additionally, there is no conformity on the diversity of fungal species in animal bedding. Thus, the diversity of results might be justified by the nature of the material used [21], their characteristics, and poultry house climate conditions [6]. Additionally, when considering the age of animals’ bedding, fungal contamination was already mentioned in both fresh and aged beds [10].

The physical and chemical parameters of chickens’ bedding were measured by one study [11]. No correlation was found between bacterial concentrations and the parameter measures (total nitrogen, ammonia nitrogen, phosphorus, potassium, solids, ash, moisture content, and pH). Despite these findings, the moisture level at chick placement seems to play an important role in the potential to host various fungal species [6]. It has been suggested that with a bird’s development, there is a higher decomposition of animals’ bedding. Along with the accumulation of fecal materials, it brings about resemblances in features of all kinds of bedding materials. This leads all the material types towards uniformity by the end of the raising period [6]. Briefly, it may seem that the type of material used on animals’ bedding does not have a significant impact on microbial counts of the crop content. However, results from other studies contradict such findings [6,10,11]. Moreover, adding new material may promote exposure to inhalable dust and fungal spores [40], partly originated by the microbial degradation of the previous straw [10].

In what concerns the sources of contamination, there is some evidence emphasizing birds as the main vector contributing to bacteria cross-contamination in animals’ bedding and water samples [40]. Feed contamination might be originated from birds’ feces that contaminated the manual hanging feeders and from chickens’ bedding due to maladjusted feeders [21]. This reason would explain the frequent contamination in feed [2,32,37,38] and drinkers’ samples [32,36,37,38]. Some suggest eggs as the initial source of *Salmonella* sp. [22]. Additionally, a high rate of fecal colonization promotes widespread bacteria cross-contamination in the poultry environment [22]. Others emphasize the role of vectors, such as rodents as an effective vector of transmission [39]. Horizontal transmission from the environment seems to be a recurrent source of *Campylobacter* sp. to broilers within a flock. Additionally, high flock size, water supplies, bedding material, fecal contact, and personnel may promote the risk of colonization and dissemination [36].

There is reasonable evidence supporting broiler manure and the material used as animals’ bedding as the main sources for inside-air microbial contamination [3]. Therefore, the bedding material can be contaminated through broiler feces, favoring pathogen transmission through the flock [36]. Indeed, cross-contamination in broiler poultry farms was already reported [42]. 

Only a few studies have demonstrated the aerosolization of animals’ bedding-associated microorganisms [10,43]. The concentration of airborne microorganisms varies greatly inside poultry buildings which might be justified by the diversity of sampling methods used in different studies, poultry species, or even building features (dimension and microclimate conditions) [3]. Hence, bioaerosol composition assessment is necessary to better understand the relationship between these factors and the impact on the health of both workers and animals [43].

Microorganisms present on chickens’ bedding are easily accumulated and aerosolized [2], being part of bioaerosols that comprise airborne bacteria, fungi, viruses, and their products such as endotoxins and mycotoxins [43]. In fact, the dose required to contract effects via the nasal route is one order of magnitude less by the respiratory tract than by ingestion which emphasizes the potency of the respiratory route [2].

Air sampling is particularly efficient when it comes to detecting “small spored” genera such as *Aspergillus* and *Penicillium* [2], the impaction method being the main mechanism for bioaerosol sampling [44], justifying the use of this technique in several articles (4 out of 19). Such a method allows the biological particles collected to remain undamaged. Thus, when providing the appropriate conditions through cultivation methods the organism must be able to form colonies, enabling identification [45]. Despite the advantages, active sampling methods are limited when it comes to assessing long-term exposure due to short time sampling of the sampling device [46]. Indeed, the variation in airborne microbial contamination it is a well-known occurrence [46,47,48].

The use of passive sampling methods enables accessing contamination levels from a large period of time [49]. The potential of surface samples has already been evidenced [50], which might be the cause behind the use of this matrix in several studies (8 out 19). The benefits of using both active and passive methods for a more accurate risk characterization are well-known [51,52]. As a matter of fact, the use of more than one type of sampling method, such as surface sampling along with air sampling, is suggested as an essential method to achieve fungal-contamination characterization and evaluation [10,53]. 

Regarding analytical methods, all studies relied on culture-based methods to perform microorganism identification. The frequent use of culture-based methods for microbial contamination assessment was recognized [43], which is essential to estimate health risks since microorganisms’ viability can restrain microorganisms’ inflammatory and/or cytotoxic potential [48,54]. However, some common microorganisms cannot be cultivated using standard techniques [55]. Additionally, conventional methods may underestimate the results obtained since incubation temperature and culture media may promote specific species [10]. Those limitations might justify the frequent use of molecular tools as an additional method in some studies (10 out of 19 studies). 

Real-time quantitative polymerase chain reaction (Q-PCR) is suggested as a precise and reliable method for bioaerosol assessment [43], overcoming culture-based methods’ limitations [56]. These methods allow the detection of non-viable microorganisms [57], as well as their components possibly having allergenic properties [58]. As already suggested, a multi-approach regarding sampling methods and laboratory assays including culture-based methods along with molecular tools should be performed in order to improve data findings [59]. 

The main findings obtained in this review enables targeting the most reported microorganisms in the poultry industry. The majority of the selected studies (16 out of 19) focused on bacterial contamination in the poultry industry. In fact, for a better bacterial identification, few performed biochemical tests (8 out of 19) and serotyped their samples (4 out of 19). Currently, typing methods are used to establish the epidemiology of bacterial infections [30]. Regarding bacteria contamination, the majority of studies evidence *Salmonella* sp. has the prevalent genera (9 out of 19). *Salmonella* is one of the major causes of foodborne infections worldwide. When improperly prepared, the consumption of poultry products previously contaminated may cause salmonellosis [30]. Birds can be asymptomatic carriers of *Salmonella*; thus, bacteria surveillance in poultry farms and good hygiene practice are important factors to prevent outbreaks of salmonellosis. 

There are several routes of microbial transmission. Chicks are very susceptible to *Salmonella* sp. Infection; consequently, microbial colonization can happen through vertical transmission or by horizontal transmission, through contaminated hatcheries, cloaca infection, transportation equipment, feed, animals’ bedding, air, uncleaned facilities, and vectors [42]. Indeed, the presence of endotoxins from bacteria in poultry aerosol was already demonstrated [33], with airborne bacteria and their metabolites increasing drastically throughout a chicken’s growth [43].

Other genera such as *Campylobacter* sp. (5 out of 19) and *Staphylococci* sp. (4 out of 19) were also recurrent, as well as *E. coli* (3 out of 19), *Enterococci* (3 out of 19) and *Listeria* sp. (3 out of 19). Despite the low frequency of *Listeria* sp. outbreaks in poultry flocks, the high prevalence in chicken farms might entail a potential risk, promoting several diseases in children, pregnant women, the immune-compromised and the elderly [21]. 

Regarding fungal assessment, *Penicillium* sp. was dominant (3 out of 19) and *Scopulariopsis* sp. and *Aspergillus* sp. were equally prevalent (2 out of 19, respectively). These findings need to be taken into consideration since some detected fungal species are considered potential agents of infection to both humans and animals [10]. *Aspergillus* section diversity (*Flavi*, *Fumigati*, *Nidulantes*, and *Terrei*) reported by one study is of the highest importance [2]. Aspergillosis is known to cause severe outbreaks in turkeys [2,41]; consequently, the low productivity results in considerable monetary loss to industries [2]. Even though *A. fumigatus* is the most common agent of *Aspergillus* infection (on humans and birds), it is not the only pathogenic species in the genus [2]. Indeed, toxigenic species such as *Aspergillus flavus* can also cause avian infections [2]. It is noteworthy that both species were already identified in animals’ bedding material [10] and air samples [2] from poultry.

The removal of animals’ bedding might involve higher exposure of poultry workers to dust, microorganisms, and their metabolites, including mycotoxins [10]. Although only one study included mycotoxins assessment, the fact that zearalenone was detected in all samples from animals’ bedding supports the idea of mycotoxins’ presence in these facilities [10,60]. As a matter of fact, in poultry facilities, occupational exposure to Aflatoxin B1, the most potent hepatocarcinogen known in mammals and produced by *Aspergillus flavus*, was already emphasized [61]. Additionally, the identification of mycotoxins producers such as *Penicillium* sp. and *Aspergillus* sp. emphasizes the need to evaluate occupational and public health risks [10]. A high level of exposure to fungi is associated with animal production facilities [60]. Fungal growth and sporulation are promoted by poor-quality bedding or contaminated feedstuffs in indoor farms. Indeed, fungal contamination has been reported not only in the poultry industry but also in swine production. Therefore, exposure to mycotoxins is a current concern in these settings [60]. 

Most of the occupational studies that focus on fungal contamination disregard the burden by their metabolites, and their possible interactions [61]. Besides the occupational health problem, the presence of mycotoxins should be considered a food safety concern and the development of studies is crucial to clarify this subject [61]. 

Currently, antimicrobial resistance is one of the major concerns regarding public health [11]. Epidemic situations such as the avian influenza in 2016, resulting in high mortality rates in flocks, may have promoted the high prophylactic use of antibiotics [4]. From the selected studies, a considerable portion evidenced bacteria resistant to more than one antibiotic class (8 out of 19). In recent years, several cases of bacterial contamination in poultry products have been reported [11,22]. Additionally, there has been an increase in resistant bacterial pathogens [38]. Thus, the recurrent frequency of this situation may have been the main reason for the development of further studies regarding this subject.

None of the selected studies performed a fungal resistance profile assessment. In fact, only some studies considered fungal contamination in these facilities (3 out of 19). These findings corroborate the lack of information regarding fungal contamination in animals’ bedding, already reported by some studies [2,10]. The frequent use of azole fungicides in agriculture and the development of fungal resistance has been described [62,63]. Recently, azole resistance associated with *Aspergillus* section *Fumigati* isolates has been found in sawmill facilities [64,65]. Such findings might be justified by the overuse of fungicides by the wood industry [65], which may represent a reservoir or resistant fungi in this setting [48,66], and in others where the wood straws are applied, such as the poultry pavilions [64]. Indeed, positive correlations were observed between wood-based material used as animals’ bedding and fungal contamination (CFU/g) in poultry pavilions [10]. 

In poultry industries, environmental conditions are usually favorable for fungal development. At the same time, birds are one of the few species capable of acquiring fungal infections without showing symptoms of illness [2]. Taking into consideration these concerns, mycoflora characterization (including the resistance profile) in these settings is essential to evaluate occupational and public health risks from fungi and their metabolites [10].

The majority of studies that included the bedding material as a source of microbial contamination in the poultry industry were performed in the United States of America. Studies’ efforts on this matter might be related to the fact that poultry is widely produced in United States [67] and accounts for about one-third of all poultry exports globally. Moreover, poultry were associated with the highest number of outbreaks illnesses. Indeed, out of poultry associated outbreaks, mostly were caused by *Salmonella enterica* and *Clostridium perfringens* [68].

Some studies already reported fungal contamination in poultry facilities being a potential reservoir of fungi [69,70,71,72]. On the other hand, the role of bedding material regarding this subject remains little-explored [6,10]. 

The presence of microorganisms in the poultry industry contributes to a potential risk of zoonosis, since microorganisms can persist along the integrated production chain leading to the contamination of the final product. This highlights a serious public health hazard [23]. Therefore, selecting a suitable bedding material may reduce the risk [12], while increasing the productive performance of birds [73]. Currently, there is no legislation on what concerns the appropriate material for animals’ bedding; thus, several kinds of substrates are used [12]. Additionally, information related to a common sampling protocol and analyses for an accurate microbial exposure assessment remains rare. 

In short, there is a lack of information concerning the impact of the bedding material used on microbial development and the health hazards that may be triggered for workers, animals, and consumers’ health. Further research concerning animals’ bedding microorganisms and toxicological potential is crucial to ensure its safety and best uses [44]. Therefore, a One Health approach is required to ensure a combination of indicators that reflect the real cost associated with microbial diseases in livestock in order to develop mitigation strategies promoting health, development, and environmental protection [1].

## 5. Conclusions

Overall, this article illustrates the microbial contamination present in poultry facilities, emphasizing animals’ bedding as a potential source of contamination. It allowed the identification of the sampling methods and assays used for microorganisms’ exposure assessment and to identify the knowledge gaps regarding microbial exposure and risk characterization. This paper should be considered when planning sampling campaigns and laboratory assays to accomplish a reliable microbial exposure assessment in poultry facilities.

Few papers were found reporting fungal exposure in poultry facilities worldwide. Moreover, less is known regarding microorganisms’ contamination on bedding materials, as well as their metabolites and possible multidrug resistance, especially when it comes to fungi. Additionally, information remains scarce concerning the expected health hazards to workers, animals, and consumers that result from microbial exposure.

Thus, in the scope of a One Health approach, a wider investigation is needed to better characterize this setting and to identify the measures to be implemented to reduce the risk and minimize the exposure.

## Figures and Tables

**Table 1:** Inclusion and exclusion criteria in the articles selected.

Inclusion Criteria	Exclusion Criteria
**Articles published in the English language**	Articles published in other languages
**Articles published from 1 January 2000 to 1 January 2022**	Articles published prior to 2000
**Articles published in any country**	
**Articles related to microbial contamination of litter in poultry production**	Articles related exclusively to litter abiotic conditions, without mentioning the microbial contamination
**Original scientific articles on the topic**	Abstracts of congress, reports, reviews/state-of-the-art articles

**Table 2:** Data selected from the chosen papers.

Database	Title	Country	Occupational Environments	Sampling Methods	Other Analysed Matrixes	Analytical Methods	Main Findings	References
PUBMED	The prevalence of Campylobacter species in broiler flocks and their environment: assessing the efficiency of chitosan/zinc oxide nanocomposite for adopting control strategy	Egypt	Poultry farms (*n* = 4)	Bedding material samples (*n* = 15)	Cloacal swabs (*n* = 100) Water (*n* = 15) Attendants’ hand swabs (*n* = 30)	Culture-based methods; morphologic identification (bacteria); biochemical tests; molecular tools (PCR).	The prevalence rate of *Campylobacter coli* in broiler poultry farms was (27.3%; 60/220). *C. coli* was detected in the highest percentage in the manure storage area and bedding material samples (66.7%; 10/15 and 53.3%; 8/15, respectively) followed by feeders, attendants’ hands, and drinkers (40.0%; 6/15, 33.3%; 5/15, 16.7%; 5/30, and 13.3%; 2/15, respectively).	[38]
	Prevalence, Concentration, and Antimicrobial Resistance Profiles of Salmonella Isolated fromFlorida Poultry Litter	USA	Poultry farms (*n* = 18)	Bedding material samples (*n* = 54) Days = 45 days Material = pine shavings	nr	Culture-based methods; morphologic identification (bacteria—*Salmonella* sp.); molecular tools/PCR; serotyping antibiotic resistance; physical and chemical parameters.	*Salmonella* was recovered from all farms (*n* = 18) with a sample prevalence of 61.1% (33/54). The prevalence and concentration of *Salmonella* recovered from animals’ bedding did not significantly differ between seasons. Overall, no correlation was found between the concentration of *Salmonella* and any of the chemical, physical, or microbial properties measured. *Salmonella* isolates (*n* = 47) tested for antimicrobial susceptibility were observed to be resistant to tetracycline (29.8%), sulfisoxazole (23.4%), and streptomycin (14.9%).	[11]
	Listeria Species in Broiler Poultry Farms: Potential Public Health Hazards	Cameroon	Poultry farms (*n* = 10)	Bedding material samples (*n* = 8)	Chicken meat carcass (*n* = 8) Feed (*n* = 2) Water (*n* = 2)	Culture-based methods; morphologic identification (bacteria—*Listeria* sp.); biochemical tests; antibiotic resistance.	*Listeria* sp. was found in 95 of 200 tested samples (47.5%), of which 42 were from animals’ bedding, 37 from raw meat, 14 from feed, and 2 from water *L. innocua* was the predominant *Listeria* sp. *L. ivanovii* was also isolated from broiler poultry farm samples and was the second most predominant (12.5%). The presence of *L. ivanovii* might be due to the nature of animals’ bedding used that is mainly from pasture-raised animals (such as wood shavings, hay, or chopped rice straw). There was a *Listeria* sp. prevalence in animals’ bedding of 52.5% (42/80). The results indicated high levels of resistance to amoxicillin/clavulanate (40%), followed by norfloxacin (38%), amoxicillin/flucloxacillin (35%), ofloxacin (32%), and ciprofloxacin (25%).	[21]
	Fungal Contamination of Poultry Litter: A Public Health Problem	Portugal	Poultry farms (*n* = 7)	Bedding material samples (*n* = 21) Weight = 10 g Days = fresh (7) and aged (14) Material = pine shavings; straw; wood shavings; rice bulls	Air samples (*n* = 27 impaction)	Culture-based methods; morphologic identification (fungi).	Twelve different fungal species were detected in fresh animal-bedding material. *Penicillium* sp. was the most frequent genus found (59.9%), followed by *Alternaria* sp. (17.8%), *Cladosporium* sp. (7.1%), and *Aspergillus* sp. (5.7%), while on the used bedding material, 19 different fungal species were detected. *Penicillium* sp. was the most frequently isolated (42.3%), followed by *Scopulariopsis* sp. (38.3%), *Trichosporon* sp. (8.8%), and *Aspergillus* sp. (5.5%). In the new bedding material, *Aspergillus fumigatus* was the most frequent species identified (32.6%) from *Aspergillus genus*, and *A. flavus* was also isolated in 9.9% of the samples. In the aged bedding material, *Aspergillus nidulantes* was the most frequent (73.4%) among the *Aspergillus* genus, but *A. fumigatus*, *A. flavus*, and *A. niveus* were also identified.	[10]
	Molecular Epidemiological Analysis and Microbial Source Tracking of Salmonella enterica Serovarsin a Preharvest Turkey Production Environment	USA	Big poultry farm (*n* = 1)	Bedding material samples (*n* = 36) Days = 2/10 and 18 weeks Material = fresh pine shavings Collection = 5 cm	Feed = 6 Drinker swabs (*n* = 36) Turkey caeca swab (*n* = 72) Air (*n* = 26, impaction) Environmental swabs (*n* = 42, walls, ventilation fans, feathers, employee shoes, feed storage, and door handles)	Culture-based methods (bacteria—*Salmonella* sp.); morphologic identification; biochemical tests.	From the 991 samples, 6% were positive for *Salmonella*. 42/145 of these were positive from animals’ bedding samples, 4/145 from feed, 24/145 from drinkers, 3/145 from leftover feed, and 12/145 from environmental swabs. The frequency of *Salmonella* detected in flocks 1, 2, and 4 was 83%, 11%, and 6%, respectively. *Salmonella heidelberg* was the most prevalent *Salmonella* serovar isolated. Overall, 79% of *Salmonella* strains were resistant to one or more antimicrobials.	[30]
	Prevalence and Antibiotic Resistance of Salmonella Isolated from a Poultry Farm and Processing Plant Environment in the State of Kuwait	Kuwait	Big poultry farm (*n* = 1)	Bedding material samples (*n* = 550) Weight = 10 g	Feed (*n* = 550/10 g) Water (*n* = 546/10 mL) Air (*n* = 72, impaction) Drinker swabs (*n* = 5) Paper tray liners (*n* = 24)	Culture-based methods (bacteria—*Salmonella* sp.); morphologic identification; serotyping.	Out of 2882 samples collected, 156 samples (5.4%) were positive for *Salmonella* sp. Contamination was 1.5% (8/550) from animals’ bedding, 0.7% (4/550) from feed, 0% (0/30) in water, 0.2% (1/546) in drinkers’ swabs, and 0% in (0/24) in paper trays. *Salmonella* was not detected in any of the paper liner, air, or water samples.	[22]
	Evolution of the Environmental Contamination by Thermophilic Fungi in a Turkey Confinement House in France	France	Small poultry farm (*n* = 1)	Bedding material samples (*n* = 124)Weight = 1 g Days = over 16 weeks Material = Fresh straw	Air (*n* = 112, Impaction) Feed (*n* = 48/1 g)	Culture-based methods (fungi); morphologic identification; molecular tools (*A. fumigatus).*	The three species that were most frequently identified were: *Absidia corymbifera* (114 samples; 40.1%), *Aspergillus fumigatus* (114 samples; 40.1%), and *A. flavus* (67 samples; 23.6*%). Scopulariopsis* sp. and *Penicillium* sp. were also regularly encountered, in addition to yeasts of the genus *Candida*. The opportunistic species *C. albicans* was detected from 195 environmental samples (68.7%). Samples obtained during the 16-week study period yielded *A. fumigatus* at 0.3 CFU/g (from 0.0 to 1.5) in animals’ bedding. After new bedding material was added at week 10, there was no isolation of fungi for 2 weeks. However, during week 14, the number of *A. fumigatus* colonies increased (1.5 CFU/g).	[2]
SCOPUS	Prevalence, biosecurity factor, and antimicrobial susceptibility analysis of Salmonella species isolated from commercial duck farms in Korea	Korea	Big duck farms (*n* = 31)	Bedding material samples (*n* = 465) Weight = 10 g	Wall swab (*n* = 186) Nipple swab (*n* = 186) Feed pan swab (*n* = 279) Dust sample (*n* = 31; 10 gr)	Culture-based methods (bacteria—*E. coli*); morphologic identification; molecular tools (PCR); serotyping; susceptibility test.	*Salmonella*-positivity rate increased up to 35.9% after the introduction of ducklings. From 4 week the detection rate decreased by 11.4%. Similarly, the actual number of *Salmonella*-positive samples was highest when the ducklings were 1–3 weeks of age, followed by when they were 4–6 weeks of age. The contamination rate was 7.5% for animals’ bedding, 3.2% for wall swabs, 3.2% for feed pan swabs, and 1.6% for dust samples. All isolates were resistant to erythromycin (194 isolates; 100%) and 122 isolates (62.9%) were resistant to nalidixic acid, followed by ampicillin (85 isolates; 43.8%), trimethoprim/sulfamethoxazole (77 isolates; 39.7%), tetracycline (74 isolates; 38.1%), cefazolin (39 isolates; 36.6%), streptomycin (39 isolates; 20.1%), and ciprofloxacin (23 isolates; 11.9%).	[39]
	Microbial Contamination of Chicken Litter Manure and Antimicrobial Resistance Threat in an Urban Area Setting in Cameroon	Cameroon	Big poultry farm (*n* = 26)	Bedding material samples (*n* = 71) Days = 26 new (in store bags) + 45 aged Material = wood shavings	nr	Culture-based methods (bacteria); morphologic identification; biochemical tests; antibiotic resistance.	*E. coli* sp. and *Salmonella* sp. were isolated in 80.8% and 36.8% of farms, respectively. 59.2% of animals’ bedding samples tested positive for *E. coli*, and 15.5% of wood shaving samples were positive for *Salmonella* sp. 28% of *E. coli* isolates were resistant to five antibiotics or more. For *Salmonella* sp., 36% were multidrug-resistant while 27% of isolates were found to be sensitive to all antibiotics tested.	[4]
	Poultry litter as potential source of pathogens and other contaminants in groundwater and surface water proximal to large-scale confined poultry feeding operations	USA	Poultry farm (*n* = 9)	Bedding material samples (*n* = 4)	nr	Culture-based methods (bacteria); morphologic identification; biochemical tests; molecular tools; mycotoxins.	Trace organic contaminants were most frequently detected in animals’ bedding. Mycotoxin compound zearalenone was detected in all animals’ bedding samples. Animals’ bedding had the largest number of microbial detections and all samples (100%) were positive for presumptive *Campylobacter* sp., Enterococci, Staphylococci, and Lactobacilli growth.	[31]
	Prevalence and genetic diversity of C. jejuni isolated from broilers and their environment using flaA-RFLP typing and MLST analysis	Lithuania	Poultry farms (*n* = 4)	Bedding material samples (*n* = 310) Days = 1/week over 2 years	Cloacae swabs (*n* = 402) Drinker swab (*n* = 50)	Culture-based methods (bacteria—*Campylobacter*); morphologic identification; molecular tools (PCR).	*Campylobacter* sp. was detected in 12 out of 13 broiler flocks (92.3%). From 1479 samples, 315 (21.3%) samples were positive for *Campylobacter* sp. *C. jejuni* was identified in 269 (85.4%) samples and *C. coli* in 26 (8.3%) samples. The highest positive samples of *Campylobacter* sp. were found in broiler cloacae, puddle water, and in animals’ bedding of additional houses.	[36]
	Tracing of Salmonella Contaminations Throughout an Integrated Broiler Production Chain in Dakahlia Governorate, Egypt	Egypt	Poultry farms (*n* = 3)	Bedding material samples (*n* = 15)	Cloacae swabs (*n* = 145) Feed (*n* = 15) Water (*n* = 15) Workers’ hand swabs (*n* = 15) Slaughterhouses’ environmental samples (*n* = 15) Samples from chicken carcasses (*n* = 15)	Culture-based methods (bacteria—*Salmonella*); morphologic identification; biochemical tests; molecular tools; serotyping.	The overall frequency of *Salmonella* contamination in the live broiler flocks was 40.9% (90/220) with a prevalence of 60% (9/15) from animals’ bedding samples, 37.9% (55/145) from cloaca swabs, 40% (6/15) in feed, 53.3% (8/15%), 20% (13/15) on workers’ hands, 60% from slaughterhouses (6/10), and 25.6% (120/30) from chicken carcasses. The isolated serovars from broiler farms were distributed as follows: *S. enteritidis* 38.8% (35/90), *S. kentucky* 23.3% (21/90), *S. typhimurium* 11.1% (10/90), *S*. molade 7.8% (7/90), *S.takoradi* 6.7% (6/90), *S. bargny* 2.2% (2/90) and 3.3% (3/90) for each of *S. papuana*, *S. tamale*, *and S. infantis.*	[23]
	MICROBIAL POLLUTION OF MANURE, LITTER, AIR AND SOIL IN A POULTRY FARM	Bulgaria	Big poultry farm (*n* = 1)	Bedding material samples (*n* = 8) Weight = 200 g Days = 40 days (first and last week)	Air samples (*n* = 4, sedimentation method Matusevich)	Culture-based methods (bacteria); morphologic identification (*E. coli*).	The number of cultivable microorganisms in animals’ bedding (logCFU/kg^−3^) varied between 6.08 and 6.92, 3.92, and 5.28 in air log CFU/m^−3^. Fresh bedding material is a source of inside and outside air and soil pollution with saprophytic microorganisms including coliform bacteria, subject to sanitary control.	[3]
	Association of Campylobacter spp. levels between chicken grow-outenvironmental samples and processed carcasses	USA	Small poultry farm (*n* = 4)	Bedding material samples	Air samples (*n* = 10, filtration), shoe coverings (*n* = 10), feed and drinker swabs (*n* = 10)	Culture-based methods (bacteria—*Campylobacter* sp.); morphologic identification; biochemical tests.	*Campylobacter* sp. was discovered in 27% (32/120) of all house samples (air, fecal/bedding material, and sponge).	[32]
	Microbial and antibiotic resistant constituents associated with biological aerosols and poultry litter within a commercial poultry house	USA	Poultry farms (*n* = 8)	Bedding material samples (*n* = 17) Weight = 10 g Collection of surface litter (0–7.5 cm)	Air (*n* = 89, impinger)	Culture-based methods (bacteria); morphologic identification; biochemical tests; molecular tools (PCR); antibiotic resistance.	Bacteria contamination was found in animals’ bedding samples with Staphylococci most likely accounting for approximately 90% of all culturable bacteria. House aerosol levels significantly increased from outside to inside the house, from approximately 6.7 × 10^3^ to 4.0 × 10^6^ CFU/m^−3^ for aerosolized heterotrophic plate count bacteria. Approximately 80% of animals’ bedding isolates were resistant to at least one antibiotic class regardless of broiler presence. However, poultry aerosol isolates’ antibiotic resistance was directly influenced by the presence of the flock. Approximately 66% (244/367) of aerosol isolates were resistant to at least one antibiotic class.	[33]
Web of Science	Risk factors related to bacterial contamination by Enterobacteriaceae and fecal coliforms and the prevalence of *Salmonella* spp. in Algerian farms, slaughterhouses and butcheries: a two-year follow-up study	Algeria	Poultry farms (*n* = 10)	Bedding material samples (*n* = 10) Weight = 5 g Days = aged	Floor and wall swabs (*n* = 20), feed (*n* = 10), water (*n* = 10).	Culture-based methods (bacteria); morphologic identification.	The highest presence of *E. coli* was observed at the poultry farms, mainly on the floors and feed (100%), bedding material (80%), floor/walls (50%), and water (20%). The contamination by *E. coli* was found in walls (100%), floors (60%), water (40%), liver and neck skin (6.66%) samples, respectively. *Salmonella* sp. were mainly isolated from neck skin (60%), liver (33.33%), walls, water, and floors (40%). The presence of *E. coli* in chicken meat was 46.66%. In addition, 28% of the chicken meat samples were contaminated with *Salmonella* sp. *E. coli* was isolated from the majority of poultry farms (70%) and *Salmonella* sp. in 22% of the poultry farms.	[37]
	Cultivation and qPCR Detection of Pathogenic and Antibiotic- Resistant Bacterial Establishment in Naive Broiler Houses	USA	Small poultry farm (*n* = 3)	Bedding material samples (*n* = 11) Weight = 100 g Days = 3 weeks Material = rice hull Collection = 10–15 cm	nr	Culture-based methods (bacteria) Morphologic identification; Molecular tools (PCR); Antibiotic resistance.	Soil levels for HPC, Staphylococci, and fecal indicators (*E. coli*, *C. perfringens*, and enterococci) were 4 × 10^6^ CFU/g^−1^, 2 × 104 CFU/g^−1^, and below detection, respectively. 100% of bedding material samples were positive for *Salmonella* sp. and *Listeria* sp. The microbial levels in the preflock were lower than typical in-use animal-bedding levels. *Salmonella* sp., *Campylobacter* sp., and *Listeria* sp. were not detected in animals’ bedding from the preflock. Surprisingly, given the overall low moisture of fresh bedding material (~90% solid content), *E. coli* and Enterococci were detected. Most bacterial isolates were resistant to at least one antibiotic class, with most isolates resistant to more than one antibiotic class.	[34]
	Spatial and temporal analysis of microbial populations in production broiler house litter in the southeastern United States	USA	Poultry farms (*n* = 8)	Bedding material samples (*n* = 16) Weight = 100 g Days = fresh and aged (0.2.4 and 6 wk) Material = fresh pine shavings	nr	Culture-based methods (Bacteria) Morphologic identification Molecular tools (PCR) Antibiotic resistance	*Staphylococcus* sp., *Enterococcus* sp., and *C. perfringens* were isolated from all locations. *Campylobacter* sp. was not detected from any sample collected. Broiler age had a significant effect on nearly all studied microbes (*p* < 0.05). *Salmonella enterica*, *L. monocytogenes*, *Enterococcus* sp., *Staphylococcus* sp., and *C. perfringens* levels were all associated with broiler age. Of the 192 samples analyzed, 28 Salmonella-positive and 47 Listeria-positive samples were identified from three flocks. *Staphylococcus* sp. were present at greater levels than any other bacteria in animal bedding samples. Most *Staphylococcus* sp. isolates (29/48) were predominantly susceptible to all tested antibiotics. *Salmonella enterica*, *Enterococcus* sp., *C. perfringens*, and *L. monocytogenes* isolates possessed multiple antibiotic resistance profiles.	[35]
	Litter mycology and the impacts of litter type and preslaughter feed withdrawal on crop bacterial community in broiler chicken	Iran	Small poultry farm (*n* = 1)	Bedding material samples (*n* = 273) Weight = 100 gr Days = 56 days Material = wood shavings (WS), cow dung (CD), shredded paper (SP), barley stalks (BS), rice husks (RH) and a mixture of identical proportionfrom all materials (Mix) Collection = 5 cm	nr	Culture-based methods (fungi); Morphologic identification	There was a significant frequency of *Mucor* sp. (14/41 rice husk), *Pencillium* sp. (17/62 cow dung), *Aspergillus* sp. (20/64 mixture of all bedding materials) *Geothrichum* sp. (18/53 shredded paper), *Monobelpharios* sp. (5/12 barley stalks), *Alternaria* sp. (1/3 rice husk, cow dung and mix) *Rhizopus* sp. (15/37 barley stalks) and *Fusarium* sp. (1/1 barley stalks) in animals’ bedding material compared to other fungi species (*p* < 0.05). The frequency of occurrence for *Aspergillus* sp. was significantly greater in the mix when compared with other materials used (*p* < 0.01).	[6]

## Supplementary Materials

The following supporting information can be downloaded at: https://www.mdpi.com/article/10.3390/ijerph192416508/s1, Table S1: Prisma checklist.
